# CONSTAX: a tool for improved taxonomic resolution of environmental fungal ITS sequences

**DOI:** 10.1186/s12859-017-1952-x

**Published:** 2017-12-06

**Authors:** Kristi Gdanetz, Gian Maria Niccolò Benucci, Natalie Vande Pol, Gregory Bonito

**Affiliations:** 1 Department of Plant Biology, Michigan State University, East Lansing, Michigan 48824 USA; 2 Department of Plant, Soil, & Microbial Sciences, Michigan State University, East Lansing, Michigan 48824 USA; 3 Department of Microbiology & Molecular Genetics, Michigan State University, East Lansing, Michigan 48824 USA

**Keywords:** taxonomy classifiers, RDP, SINTAX, UPARSE, UNOISE, ITS, mycobiome, fungal microbiome

## Abstract

**Background:**

One of the most crucial steps in high-throughput sequence-based microbiome studies is the taxonomic assignment of sequences belonging to operational taxonomic units (OTUs). Without taxonomic classification, functional and biological information of microbial communities cannot be inferred or interpreted. The internal transcribed spacer (ITS) region of the ribosomal DNA is the conventional marker region for fungal community studies. While bioinformatics pipelines that cluster reads into OTUs have received much attention in the literature, less attention has been given to the taxonomic classification of these sequences, upon which biological inference is dependent.

**Results:**

Here we compare how three common fungal OTU taxonomic assignment tools (RDP Classifier, UTAX, and SINTAX) handle ITS fungal sequence data. The classification power, defined as the proportion of assigned OTUs at a given taxonomic rank, varied among the classifiers. Classifiers were generally consistent (assignment of the same taxonomy to a given OTU) across datasets and ranks; a small number of OTUs were assigned unique classifications across programs. We developed CONSTAX (CONSensus TAXonomy), a Python tool that compares taxonomic classifications of the three programs and merges them into an improved consensus taxonomy. This tool also produces summary classification outputs that are useful for downstream analyses.

**Conclusions:**

Our results demonstrate that independent taxonomy assignment tools classify unique members of the fungal community, and greater classification power is realized by generating consensus taxonomy of available classifiers with CONSTAX.

**Electronic supplementary material:**

The online version of this article (10.1186/s12859-017-1952-x) contains supplementary material, which is available to authorized users.

## Background

Next-generation sequencing technologies and high-performance computers define the culture-independent era of microbial ecology. High-throughput sequencing of DNA barcode marker regions, namely the bacterial 16S rRNA gene or fungal internal transcribed spacer (ITS) ribosomal regions, have allowed researchers to characterize complex microbial communities at a depth not previously possible with culture-based methods. Hypervariable regions of the 16S rRNA gene have been extensively studied and adopted by researchers to describe prokaryotic microbial communities, and a mix of ribosomal markers have been used to describe fungal communities [1] over the past 25 years [2]. The ITS region, comprising the ITS1, 5.8S, and ITS2 segments, was recently selected as the formal DNA barcode for fungi [3–5], although there is a lack of consensus regarding which ITS (ITS1 or ITS2) to utilize as a barcode [6–8]. It remains unclear which of the ITS primer sets has the best resolution for fungal diversity, and papers targeting either ITS segment have been published at near equal frequencies [8–10].

Pipelines for processing fungal ITS amplicon datasets such as CLOTU [11], CloVR-ITS [12], PIPITS [1], and others [13] are available in the literature, but most of the tool-development effort has been towards generating nearly automated pipelines for filtering, trimming, and clustering of amplicon reads into operational taxonomic units (OTUs). Less emphasis has been placed on assigning taxonomy to representative OTU sequences in a dataset. Linnaean taxonomy provides a controlled vocabulary that communicates ecological, biological or geographic information. Linking OTUs to functionally meaningful names, which typically depends upon species-level resolution, is key to addressing biological and ecological hypotheses. Processing sequencing reads, in addition to taxonomy assignment of sequences, can be completed using various bioinformatics pipeline tools. The most popular are Mothur [14], QIIME [15], and USEARCH [16]. There are a variety of algorithms to use for the taxonomy assignment step, which include: BLAST [17], Ribosomal Database Project (RDP) Naïve Bayesian Classifier [18], UTAX [19], and SINTAX [20]. The RDP Classifier (RDPC) uses Bayesian statistics to find 8-mers that have higher probability of belonging to a given genus. Based on these conditions, RDPC estimates the probability that an unknown query DNA sequence belongs to the genus [18]. The UTAX algorithm looks for k-mer words in common between a query sequence and a known reference sequence, and calculates a score of word counts. The score is used to estimate confidence values for each of the taxonomic levels, which are then trained on the reference database to give an estimate of error rates [19]. The SINTAX algorithm predicts taxonomy by using k-mer similarity to identify the top hit in a reference database, and provides bootstrap confidence for all ranks in the prediction [20]. Local alignment, most commonly implemented in BLAST [17], is still occasionally used for taxonomy assignment of high-throughput sequence datasets. However use of BLAST to identify OTUs in amplicon-based microbiome datasets has low accuracy as demonstrated previously [20–22], and discussed by Wang et al. [18].

The UNITE reference database is a curated database of all International Nucleotide Sequence Database Collaboration (INSDC) fungal sequences, and is the most commonly used reference database for fungal amplicon analyses [23–25]. Recently the Ribosomal Database Project released the Warcup Fungal Database [26], a curated version of UNITE and INSDC. Apart from previously published database comparisons which showed the accuracy of UNITE [23] and Warcup fungal databases [26], all comparative studies of taxonomy classifiers of which we are aware, have analyzed only prokaryotic organisms [22, 27, 28]. Since only a small fraction of microbial species estimated to be on the planet have been described, taxonomic classification is not a trivial task and no algorithm is 100% precise. Several types of classification errors are possible, as highlighted in Table 1. The RDPC, UTAX, and SINTAX classifiers report a confidence value for the classification given to an OTU so that the user can set a cutoff value below which no name is given. Even though a number of databases and tools have been developed to enable high-throughput analyses of environmental sequences, researchers still need to solve the problems caused by misidentified or insufficiently identified sequences [5]. Further, some poorly sampled fungal lineages reduce the ability of a classifier to confidently assign OTUs to the correct fungal lineage regardless of the classification algorithm used.

**Table 1:** Types of classifications

Present in the database?	Taxon name given?	Correct name given?	Result	Error Type
Yes	Yes	Yes	Good assignment	True positive
Yes	Yes	No	Misclassification	False positive
Yes	No	No	Underclassification	False negative
No	Yes	No	Overclassification	False negative
No	No	No	Good assignment	True negative

This study tested whether established taxonomic classifiers for fungal ITS DNA sequences generate similar profiles of the fungal community. Specifically, we compared the power (proportion of assigned OTUs at a given level) and consistency (agreement of OTU assignment across classifiers) of the RDPC, UTAX, and SINTAX classification algorithms. Power and consistency were compared across i) ITS1 or ITS2 regions, ii) OTU-clustering approaches, and iii) merged or single stranded reads. Further, we created a Python tool that functions independently of OTU-picking method to merge taxonomy assignments from multiple classifier programs into an improved consensus taxonomy, and generates several output files that can be used for subsequent community analysis.

## Methods

### Data accessibility

**Fig. 1:**
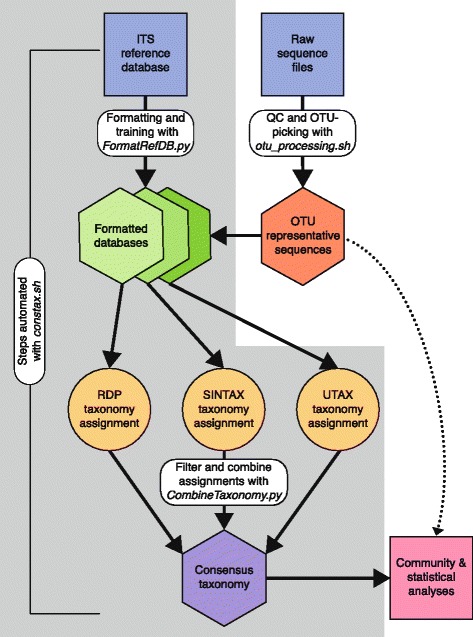
Overview of CONSTAX workflow. Bubbles highlighted by gray box are automated through *constax.sh*

Sample origins, barcode regions, and accession numbers for all datasets used in the current study can be found in Table 2. Implementation of the tool presented in this paper requires users to download and install the following software: RDPC [https://github.com/rdpstaff/classifier], USEARCH version 8 for UTAX, and USEARCH version 9 or later for SINTAX [http://drive5.com/usearch/download.html], R v2.15.1 or later [https://www.r-project.org], Python version 2.7 [https://www.python.org]. Detailed installation and analysis instructions, including all custom scripts used in the analysis and a test dataset are available in Additional file 1, or for download from GitHub: [https://github.com/natalie-vandepol/compare_taxonomy]. All of the custom Python scripts described in the methods section can be downloaded from the *CONSTAX.tar.gz* file (Additional file 2). All the steps described in the methods section are automated through the *constax.sh* script, but are included as independent scripts in *CONSTAX.tar.gz* so they can be easily modified to suit the user’s needs. An overview of the data analysis workflow is available in Fig. 1.

**Table 2:** Sample origins, barcode regions, and accession numbers for datasets

Dataset	Gene Region	Read Type	Sample Origin	Data Availability	Reference
ITS1-Soil	ITS1	2 × 250 bp	North American soil	NCBI SRA SRP035367	Smith & Peay [36]
ITS2-Soil	ITS2	2 × 250 bp	North American soil	NCBI SRA SRR1508275	Oliver et al. [37]
ITS1-Plant	ITS1	2 × 250 bp	European plants	MG-RAST 13322	Agler et al. [10]
ITS2-Plant	ITS2	2 × 250 bp	European plants	MG-RAST 13322	Agler et al. [10]
ITS1-BC^a^	ITS1	1 × 300 bp	North American soil	NCBI SRA SRP079401	Benucci et al., unpublished
ITS2-BC^a^	ITS2	1 × 300 bp	North American soil	NCBI SRA SRP079401	Benucci et al., unpublished
ITS1-UN^b^	ITS1	1 × 300 bp	North American soil	NCBI SRA SRP079401	Benucci et al., unpublished
ITS2-UN^b^	ITS2	1 × 300 bp	North American soil	NCBI SRA SRP079401	Benucci et al., unpublished

^a^ data processed with UPARSE algorithm, OTUs generated with clustering

^b^ data processed with UNOISE algorithm, ESVs generated with splitting

### Generation of operational taxonomic units

For the ITS1-soil and ITS2-soil datasets (Table 2), forward and reverse reads were merged with PEAR version 0.9.8 [29]. Merged reads were randomly sampled to one million reads to reduce computational time. Reads were quality-filtered, trimmed, dereplicated, clustered at 97% similarity (the standard sequence similarity value), and OTU-calling was performed using USEARCH version 8.1.1831 [16]. Analysis of plant datasets (ITS1-plant and ITS2-plant) began with the processed 97% similarity OTUs provided by the authors [10].

For the ITS1/2-BC and ITS1/2-UN datasets, reads were quality-filtered as above, but differed in OTU-generation method. First, a clustering algorithm that generated OTUs using the UPARSE [19] algorithm was used to call OTUs for ITS1-BC and ITS2-BC. Second, the UNOISE2 algorithm [30] that performed denoising and generated exact sequence variants (ESVs) [31] was used for ITS1-UN and ITS2-UN. Each set of OTUs and ESVs were randomly sampled to 500 for the comparative taxonomic analysis described in the next section. Sample and abundance data were not used in this study. The code for the OTU-picking pipeline described above is available in Additional file 3.

### Database formatting and classifier training

The UNITE fungal database [23], release 31–01-2016, containing 23,264 sequences was used in the current study. A custom script (*FormatRefDB.py*) was developed in Python 2.7 to format the database, starting from the general fasta release, for each classifier to ensure training was completed with identical databases. For RDPC training, custom Python scripts (*subscript_lineage2taxonomyTrain.py*, *subscript_fasta_addFullLineage.py*) were used to give each Species Hypothesis a unique name and remove special text characters. Prior to UTAX training and SINTAX classification, custom Python scripts were used to make minor changes to header lines of the *fasta* file. After formatting, these versions of the UNITE database were used to train classifiers. All the formatting and training scripts above are automated through the *constax.sh* script, users need only specify the location of the reference database.

### Taxonomy assignment

Taxonomy was assigned to the OTUs with RDPC version 11.5 [18, 32], UTAX from USEARCH version 8.1.1831 [19, 33], and SINTAX from USEARCH version 9.2 [16]. This step generated three tables (one from each classifier) with a taxonomic assignment at each of the seven ranks of the hierarchy (Kingdom, Phylum, Class, Order, Family, Genus, Species). We used the default settings, a 0.8 cut-off, to serve as a baseline for comparison. Researchers may choose to use less stringent cut-offs, depending on the goals of their studies. The cut-off can be specified in the *config* file contained in *CONSTAX.tar.gz* (Additional file 2).

### Post-taxonomy data processing

A custom Python script (*CombineTaxonomy.py*) was developed to standardize the taxonomy table formats, filter the output files at the recommended quality score, and create the consensus taxonomy. Additionally, the script produces a combined and improved (higher power) taxonomy table by concatenating the information contained in the taxonomy tables from RDPC, UTAX, and SINTAX. Rules developed to merge the taxonomy assignments implemented in the Python script are detailed in Table 3. Briefly, a majority rule (two out of three OTUs classified) was used when classifiers did not assign the same name to a representative sequence. When there was not a clear majority rule, the name with the highest quality score was chosen. The *CombineTaxonomy.py* script is also automated through the *constax.sh* script*.* All analyses downstream of the consensus OTU assignments were completed in R version 3.3.2 [34] and graphs were generated with the R package ‘*ggplot2’* [35]. R code used to generate the graphs is also available in the *CONSTAX.tar.gz,* and automated through *constax.sh* script.

**Table 3:** Rules adopted to generate the combined taxonomy table

RDP	UTAX	SINTAX	CONSENSUS
3 taxonomy assignments			
*Taxon A*	*Taxon A*	*Taxon A*	*Taxon A*
*Taxon A*	*Taxon A*	*Taxon B*	*Taxon A*
*Taxon A*	*Taxon B*	*Taxon C*	Use score
2 taxonomy assignments +1 unidentified			
*Taxon A*	*Taxon A*	Unidentified	*Taxon A*
*Taxon A*	*Taxon B*	Unidentified	Use score
1 taxonomy assignment +2 unidentified			
*Taxon A*	Unidentified	Unidentified	Taxon A

## Results

### Power of classifiers

**Fig. 2:**
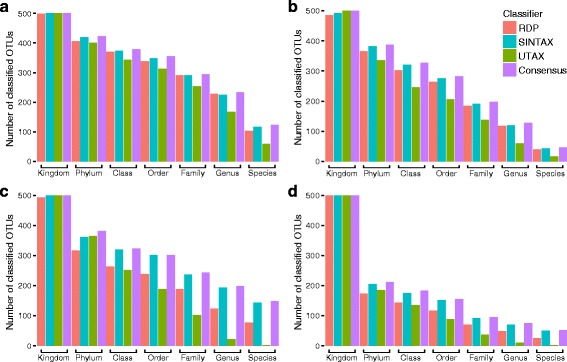
Power of classifiers. Distribution of classified and unclassified OTUs for each classifier and across taxonomic level. **a** ITS1-soil dataset from Smith & Peay [36]. **b** ITS2-soil dataset from Oliver et al. [37]. **c** ITS1-plant and **d** ITS2-plant datasets from Angler et al. [10]

Classification power differed across RDPC, UTAX, and SINTAX (Fig. 2). Also, the total number of assigned OTUs varied across datasets, ITS region, and OTU-generation approach. In general, the highest number of assignments at each level of the taxonomic hierarchy was observed in the ITS1-soil dataset shown in Fig. 2a [36]. Classifications for the ITS2-soil dataset [37] follow the same general pattern as ITS1-soil, but overall had lower power (Fig. 2b). Although, UTAX had higher classification power for some ITS1 datasets at Kingdom level (Fig. 2c, Additional file 4: Figure S1A), generally, SINTAX had the highest classification power (Fig. 2a-b, d). ITS1-plant (Fig. 2c) and ITS2-plant (Fig. 2d) [10] datasets generated a greater number of unidentified OTUs by all three of the classifiers when compared with the soil datasets (Fig. 2, Additional file 4: Figure S1). A larger number of identified OTUs were detected for the ITS1-BC and ITS2-BC datasets when OTUs were generated by denoising (Additional file 4: Figure S1A-B) instead of clustering (Additional file 4: Figure S1C-D), at all levels except Species. Moreover, a similar pattern was observed with the ITS1-BC and ITS2-BC datasets, more assigned OTUs were observed for ITS2-BC in comparison to ITS1-BC, but not at every rank level (Additional file 4: Figure S1).

Depending on the dataset, the number of unidentified OTUs gradually, or sharply, increased at other ranks higher than Kingdom level. Percent improvement of the consensus taxonomy assignments were calculated from maximum and minimum numbers of classifications obtained at any given rank (Table 4). With CONSTAX, there was ~1% mean improvement at Kingdom level when the consensus taxonomy was compared with an individual classifier program. At other rank levels, there was 7–35% mean improvement. For ITS2 datasets, there was a 1–61% percent improvement at Family level (Table 4). For ITS1 datasets there was a 1 to 59% improvement at Family level (Table 4). At Species level there was a 35% mean improvement across all datasets (Table 4). The higher end of these ranges is due to poor classification of OTUs, especially ITS2 OTUs, using UTAX. If the percent improvement is recalculated without UTAX the maximum percent improvement drops from 98% to 52% (Table 4).

**Table 4:** Range of percent improvement using CONSTAX

Taxnomic Rank^a^	Percent Increase^b^	ITS1-Soil	ITS2-Soil	ITS1-Plant	ITS2-Plant	ITS1-BC^c^	ITS2-BC^c^	ITS1-UN^c,d^	ITS2-UN^c,d^	Mean Increase
Kingdom	max.	0.00	0.00 (1.60)	0.00 (0.20)	0.00	0.00 (1.00)	0.00 (0.20)	0.00 (2.20)	0.00	0.81 (1.14)
	min.	0.40	3.00	1.40	0.20	3.60	1.40	2.60	0.40	
Phylum	max.	0.47	1.29	4.46 (5.25)	2.84	6.05	1.70	5.57 (7.62)	2.76	6.83 (6.50)
	min.	5.21 (4.03)	13.18 (5.43)	17.06	18.01	11.46	12.24	11.73	8.90	
Class	max.	1.58	1.83	0.93	4.89	3.04	0.84	3.98	3.70	8.56 (5.36)
	min.	9.23 (2.11)	24.77 (7.65)	21.98 (18.27)	26.63 (22.28)	18.26 (8.70)	9.28 (5.49)	13.94 (5.98)	9.26 (5.19)	
Order	max.	1.69	2.47	0.33	2.58	2.48	1.40	5.75	3.23	10.94 (5.73)
	min.	11.83 (4.79)	27.21 (6.71)	37.42 (20.86)	42.58 (24.52)	19.31 (7.92)	7.44 (5.58)	19.91 (8.85)	11.29 (4.03)	
Family	max.	1.36	3.54	2.47	3.16	1.86	1.10	6.88	6.13	15.72 (6.43)
	min.	13.9 (1.69)	30.81 (6.57)	58.02 (22.63)	61.05 (26.32)	20.50 (9.94)	11.60 (6.08)	32.80 (8.99)	27.83 (7.08)	
Genus	max.	2.56	6.25	2.51	5.33	2.40	1.89	9.15	9.03	27.06 (9.04)
	min.	28.21 (3.85)	53.13 (8.59)	88.94 (37.69)	85.33 (36.00)	31.20 (7.20)	35.22 (10.69)	63.38 (10.56)	62.58 (9.03)	
Species	max.	5.65	8.51	2.70	1.92	3.19	1.83	9.28	13.68	34.65 (13.20)
	min.	52.42 (16.13)	65.96 (14.89)	98.65 (47.97)	96.15 (51.92)	41.49 (9.57)	51.38 (21.10)	81.44 (11.34)	89.47 (17.89)	

^a^Percent improvement calculated with RDP, SINTAX, and UTAX outputs (numbers in paranthesis calculated without including UTAX, only differing values displayed). Ranges represent minimum and maximum improvement when compared to all three classifiers at a given level

^b^Equation to calculate percent increase, where *N* = assigned OTUs. $\frac{\max\ or\ \min\ N}{consensus\ N}\times 100$

^c^Reads are forward (ITS1) or reverse (ITS2), not merged read pairs

^d^Dataset was processed with denoising instead of clustering

### Consistency of classifiers

Generally, all the classifiers were consistent in OTU assignments. Based on the consensus taxonomy tables, no bias was observed toward a fungal lineage from any of the classifiers. Nearly all OTUs were identified at Kingdom level (Table 5, Additional file 5: Table S1). There were few examples across the datasets where a single OTU was placed into a unique lineage by one or more of the classifiers. Only 1.24% ∓ 0.006 (st. dev.) of OTUs were differentially assigned across the datasets. This differential assignment phenomenon was most frequently observed at Kingdom level where OTUs were placed with low confidence into either Kingdom Fungi or Protista (Table 5). These OTUs were rarely assigned at a higher level after Kingdom, and never higher than Class; they may be novel sequences, PCR, or sequencing errors. Across all datasets used in the present study (4000 OTUs/ESVs), there were two examples of OTUs assigned to unique fungal lineages. These were found only in ITS1-BC and ITS2-BC datasets (Table 5). The ITS1-BC OTU diverged at Class; the OTU was assigned to Eurotiomycetes and Sordariomycetes by RDPC and UTAX, respectively, and unidentified by SINTAX. This OTU did not have an assignment lower than family. The assignment of the ITS2-BC OTU diverged at Phylum; RDPC and SINTAX placed the ITS2-BC OTU into the Basidiomycota, and UTAX placed this OTU in the Ascomycota. The assignment diverged again at Class, where it was placed into the Pucciniomycetes by RDPC, and the Agaricomycetes by SINTAX.

**Table 5:** Distribution of identically classified, uniquely classified, and unidentified OTUs across all taxonomic ranks for data presented in Fig. 2

ITS1-Soil	Kingdom	Phylum	Class	Order	Family	Genus	Species
3 classified, identical	498	393	339	306	253	167	57
3 classified, 1 unique	0	0	0	0	0	0	0
3 classidied, 3 unique	0	0	0	0	0	0	0
2 classified, identical	2	17	31	33	34	53	42
2 classified, unique	0	0	0	0	0	0	0
1 classified	0	12	9	16	8	14	25
RDP	0	0	1	1	5	9	7
SINTAX	0	11	5	12	3	5	18
UTAX	0	1	3	3	0	0	0
Unidentified	0	78	121	145	205	266	376
ITS2-Soil							
3 classified, identical	481	332	242	203	135	60	16
3 classified, 1 unique	3	0	0	0	0	0	0
3 classified, 3 unique	0	0	0	0	0	0	0
2 classified, identical	2	33	58	57	45	49	20
2 classified, unique	7	0	0	0	0	0	0
1 classified	7	22	27	23	18	19	11
RDP	0	3	4	4	6	8	4
SINTAX	7	17	1	17	11	11	7
UTAX	0	2	22	2	1	0	0
Unidentified	0	113	173	217	302	372	453
ITS1-Plant							
3 classified, identical	490	304	234	181	98	22	2
3 classified, 1 unique	2	0	0	0	0	0	0
3 classified, 3 unique	0	0	0	0	0	0	0
2 classified, identical	4	52	45	65	88	97	71
2 classified, unique	4	0	0	0	0	0	0
1 classified	0	25	44	56	57	80	75
RDP	0	2	1	0	5	5	4
SINTAX	0	9	41	0	51	75	71
UTAX	0	14	2	1	1	0	0
Unidentified	0	119	177	198	257	301	352
ITS2-Plant							
3 classified, identical	499	166	120	83	36	11	2
3 classified, 1 unique	0	0	0	0	0	0	0
3 classified, 3 unique	0	0	0	0	0	0	0
2 classified, identical	1	20	29	36	32	33	22
2 classified, unique	0	0	0	0	0	0	0
1 classified	0	25	35	36	27	31	28
RDP	0	1	2	2	3	4	1
SINTAX	0	3	31	32	0	28	27
UTAX	0	21	2	2	24	0	0
Unidentified	0	289	316	345	405	425	448

### Python tool outputs

CONSTAX is implemented in Python and provided as a Bourne Shell executable, *constax.sh*. After installation of the required dependencies, the user must modify paths and parameters in *constax.sh* and the *config* file, both of which can be found in *CONSTAX.tar.gz* (Additional file 2). The Python scripts called by *constax.sh* are provided independently and can be easily modified for use with other classifiers or reference databases. After implementation of *constax.sh*, filtered versions of all taxonomy tables for the given cutoff are generated, alongside the four main output files: i) *consensus_taxonomy.txt*, the final higher power taxonomy table; ii) *combined_taxonomy.txt*, which is a large table of all three taxonomy tables side-by-side in addition to the consensus taxonomy; iii) *otu_taxonomy_CountClassified.txt*, which details assigned and unidentified OTUs at each rank level; and iv) *Classification_Summary.txt*, which lists the total counts of all unique taxa at a given rank level.

## Discussion

Factors that influence the composition and structure of microbial communities are mainly confined to three different groups: sample origin (e.g., soil or water), laboratory methods (e.g., primer selection, PCR conditions, library preparation), and post-sequencing bioinformatic analysis. Since there are sample or methodological challenges at several steps of microbial community studies that can ultimately influence taxonomic classification; we standardize and improve the taxonomic classification step of fungal microbiome studies with CONSTAX. CONSTAX improves taxonomy assignment of fungal OTUs regardless of the strategies researchers choose to reduce the sample or methodological challenges. Linking OTUs to functionally informative names, which largely requires genus- or species-level resolution, is key to addressing biological and ecological hypotheses in fungal community studies. Considerable time should be invested into choosing optimal tools for taxonomic analysis. In this study, eight fungal amplicon datasets were assigned taxonomy using the same reference database [23] and three taxonomy assignment programs were compared: RDPC [18, 32], UTAX [19, 33], and SINTAX [20]. The taxonomic classification step is arguably one of the most delicate steps of the pipeline for amplicon-based microbial ecology studies, because taxon names are largely the basis by which scientists attach biological interpretation to the data. Our results showed minor differences across taxonomic classification approaches using thresholds chosen a priori.

The UTAX classifier generated greater numbers of unidentified OTUs compared with RDPC and SINTAX, a pattern that is pronounced in the ITS2 dataset. We also found more non-fungal OTUs were recovered from the ITS2 sequences; indicating primers for this region may be less fungi-specific than those used for amplifying the ITS1 region. The ITS1 region has been shown to be more conserved in sequence and length for most fungal lineages compared with ITS2 [38–40]. Whether the ITS1 or ITS2 region provides the best taxonomic resolution has been investigated previously with Sanger sequence data [3, 37] and pyrosequence data [9, 41]. Apart from the small bias of ITS1 against early diverging fungi, these regions yield similar profiles of fungal communities and either region is considered suitable for community studies. Regardless of primer choice, we showed that use of multiple taxonomy assignment algorithms resulted in consistent classifications when an appropriate OTU-clustering threshold level is used.

Our tool, CONSTAX, implements the following best practice tips for taxonomy assignment of ITS datasets: i) Use more than one classifier program, as not one is clearly superior to others; ii) Obtain a consensus taxonomy after running multiple classifiers; iii) Use the most recent release of software. The classifier programs tested here differ slightly in power, so performing taxonomic classifications with multiple programs, and combining the results will result in a stronger assignment with higher resolution.

When designing experiments, it behooves researchers to carefully consider their target organisms when choosing the ITS barcode region and selecting primers. When investigating broad patterns of fungi, use of ITS alone should be sufficient, but if there is interest in a specific group of fungi, additional markers for those lineages (such as 18S rRNA gene for arbuscular mycorrhizal fungi) may be needed [42]. Further, there are limitations in making functional inferences from fungal ITS amplicon data. If the research questions are aimed at specific species or functions, metagenomics may be a more appropriate approach than amplicon-based community analyses.

## Conclusion

We provide a tool, CONSTAX, for generating consensus taxonomy of targeted amplicon sequence data, and demonstrate that it improves taxonomy assignments of environmental OTUs. Taxonomic assignment will improve as database completeness improves, especially the RDPC, since that algorithm functions best when there are multiple representatives for a group (genus or species). The mycological community should continue to generate high quality ITS reference sequences for their research organisms and from Herbarium specimens, which will further enhance the performance of taxonomy assignment algorithms.

## Additional files

Additional file 1: CONSTAX tutorial. Implementation of code and scripts for database formatting and trimming, taxonomy assignment, and post-taxonomy assignment filtering. (PDF 699 kb)

Additional file 2: CONSTAX.tar.gz compressed directory. Contains test datasets, Python, Shell, and R scripts to use the tool. (GZ 175 kb)

Additional file 3: otu_processing.sh pipeline. Contains code for sequence quality control and OTU-picking. (SH 2 kb)

Additional file 4: Figure S1. Power of taxonomy classifiers. Distribution of classified and unclassified OTUs for each classifier and across taxonomic level. (A) ITS1-UN and (B) ITS2-UN data analyzed using UNOISE. (C) ITS1-BC and (D) ITS2-BC data analyzed with UPARSE. (PDF 304 kb)

Additional file 5: Table S1. Distribution of identically classified, uniquely classified, and unidentified OTUs across all taxonomic ranks for data presented in Additional file 4: Figure S1 (Benucci et al., unpublished). (XLSX 47 kb)

## Abbreviations

ESV: exact sequence variant
ITS: internal transcribed spacer region of the ribosomal DNA
OTU: operational taxonomic unit
PCR: polymerase chain reaction
RDP: Ribosomal Database Project
RDPC: Ribosomal Database Project classifier
rRNA: ribosomal RNA
