# Supplementary material for: CONSTAX: a tool for improved taxonomic resolution of environmental fungal ITS sequences
Source: BMC Bioinformatics. 2017 Dec 6;18:538. doi: 10.1186/s12859-017-1952-x (PMC5719527; doi:10.1186/s12859-017-1952-x)
Supplement: Supplementary file 1 — CONSTAX tutorial. Implementation of code and scripts for database formatting and trimming, taxonomy assignment, and post-taxonomy assignment filtering. (PDF 699 kb) [file 12859_2017_1952_MOESM1_ESM.pdf]

CONSTAX: a tool for improved taxonomic resolution of environmental fungal ITS sequences

Supplemental File 1

Gdanetz, Benucci, VandePol, Bonito

The analysis requires four general steps to go from raw sequences to a consesus taxonomy:

1. Sequence QC and OTU-picking
2. Database formatting and training
3. Taxonomy assignment
4. Post-taxonomy-assignment processing, filtering, analyzing

Steps 2 through 4 are automated through *constax.sh* and implemented in the CONSTAX tool.

CONSTAX Tutorial

Step 1: Sequence QC and OTU-picking.

Use of CONSTAX assumes familarity with OTU-processing pipelines used in amplicon-sequence based microbial community studies. The QC and OTU-picking steps provided here are for reproducibility using the test datasets. If beginning with raw sequence files, follow the OTU-picking steps below. If beginning with OTUs, skip to Step 2. The code for QC and OTU-Picking is availble in Supplemental File 3.

Required Programs:

- Python version 2.7
- PEAR version 0.9.8
- USEARCH version 8 and 9/10
- RDP version 11
- R and/or RStudio

Run the Code:

```
# get quality statistics
fastqc ./*_R1_001.fastq
fastqc ./*_R2_001.fastq

# merge forward and reverse reads, change labels
```

```
usearch8 -fastq_mergepairs *_R1.fq -relabel @ -tabbedout merged_tabbed.txt -r
eport merged_summary.txt -fastqout merged.fastq

# subsample reads
# download from https://github.com/lh3/seqtk
seqtk sample -s100 merged.fastq 500000 > sub.merged.fastq

# quality filter
usearch8 -fastq_eestats2 sub.merged.fastq -output merged.pre_filtered.eestats
2.txt -length_cutoffs 100,400,10
usearch8 -fastq_filter sub.merged.fastq -fastq_minlen 150 -fastq_maxee 0.5 -f
astaout merged.filtered.fa -fastaout_discarded merged.no_filter.fa -fastqout
merged.filtered.fastq
usearch8 -fastq_eestats2 merged.filtered.fastq -output merged.post_filtered.e
estats2.txt -length_cutoffs 150,400,10

# trim cleaned reads
usearch8 -fastx_truncate merged.filtered.fastq -padlen 250 -trunclen 250 -fas
taout cleaned.fa

# find unique/representative sequences
usearch8 -derep_fulllength cleaned.fa -sizeout -fastaout derep.fa -threads 4

# cluster OTUs, remove singletons, de novo chimera check
usearch8 -cluster_otus derep.fa -minsize 2 -sizein -sizeout -relabel OTU_ -ot
us derep.otus.fa -uparseout reads.derep.otus.txt

# reference-based chimera check
# use for ITS1 region: uchime_sh_refs_dynamic_develop_985_01.01.2016.ITS1.fa
# use for ITS2 region: uchime_sh_refs_dynamic_develop_985_01.01.2016.ITS2.fa
# wget https://unite.ut.ee/sh_files/uchime_reference_dataset_01.01.2016.zip
usearch8 -uchime_ref derep.otus.fa -db /path_to_files/UNITE_db/uchime_sh_refs
_dynamic_develop_985_01_01_2016_ITS2.fa -nonchimeras otus.no_chimera.fa -uchi
meout reads.derep.otus.no_chimera.uchime -strand plus -sizein -sizeout

# map reads back to OTUs and create the otu_tab.txt for phyloseq
usearch8 -usearch_global merged.filtered.fa -db otus.no_chimera.fa -strand pl
us -id 0.97 -top_hit_only -otutabout otu_table.txt -sizein -sizeout

# subsample OTUs to 500
seqtk sample -s100 otus.no_chimera.fa 500 > OTU_500.fa
```

Step 1 Output Files:

- fasta file with all OTUs (*otus.no_chimera.fa*)
- subsetted fasta file (*OTU_500.fa*). This small fasta file for each dataset used in the paper is provided in *otus/test_data/*

Step 2: CONSTAX set-up and installation.

Download the CONSTAX.zip from GitHub or Supplemental File 2:

This zipped directory contains everything needed to run the tool. The ITS1-BC dataset has been included for users to test the tool. Users should place a copy of their own datasets within the *otus* directory.

Obtain a copy of the UNITE fungal database:

Place a copy the UNITE general release fasta (or other reference database) inside the *DB* directory.

Download UNITE database here

Install the RDP Classifier:

Will require installation of Apache Ant as detailed in the *README.txt* inside the CONSTAX directory.

RDP install instructions here

Set symbolic links for USEARCH:

Download USEARCH here

Download USEARCH8 (for UTAX) and USEARCH9/USEARCH10 (for SINTAX). Make sure to select the appropriate version for your operation system (MacOS or Linux). Place USEARCH binaries inside CONSTAX directory.

```
# change permissions for USEARCH binaries

chmod +x usearch10_0_240_i86osx32
chmod +x usearch8_1_1861_i86osx32

# create symbolic links by specifying the full paths of the executables

ln -sf /path-to-folder/CONSTAX/usearch10_0_240_i86osx32 usearch10
ln -sf /path-to-folder/CONSTAX/usearch8_1_1861_i86osx32 usearch8
```

Run the Scripts:

After installations of all the classifier programs, the tool is almost ready to use. The last step is to update the paths in the *config* file - specificallly, the location of the UNITE database, and the *otu.fasta* file. Within the *constax.sh* file, users need to update the path for the *config* file, the location of the installtion of the RDP Classifier, and the *rRNAClassifier.properties* file (part of the RDP Classifier installation).

To use CONTAX, type the following into the terminal:

```
sh constax.sh
```

As outlined in Figure 1, the *constax.sh* shell script automates several python scripts that format the reference database, train the classifiers, assign taxonomy, then generate the consensus taxonomy.

constax.sh Output Files:

Each run of *constax.sh* will create three directories within the CONSTAX directory: *training_files*, *taxonomy_asssignments*, *outputs*.

1. The *training_files* directory will contain logs and other property files to use the classifiers.
2. The *taxonomy_assignments* directory will contain the un-formatted taxonomy assignment from each classifier.
3. The *outputs* directory will contain *consensus_taxonomy.txt* (**this is the improved taxonomy assignment we recommend using**), *combined_taxonomy.txt* (this is a large taxonomy table that allows users to see how the consensus taxonomy was generated). The *outputs* directory will also contain filtered taxonomy tables for the individual classifiers; and *CountClassified.txt* and *Classification_Summary.txt* which provide statistics on the consensus taxonomy.

Step 3: Generate barplots with R.

The R code below is also called by *constax.sh* and will generate a barplot similar to those shown in Figure 2 using the *CountClassified.txt* file.

```
if(!require(ggplot2)){
  install.packages("ggplot2")
  library(ggplot2)
}

comb_tax = read.table("outputs/combined_taxonomy.txt", header=TRUE, row.names
=1, sep="\t")
system.time(comb_tax[comb_tax==''|comb_tax==' ']<-NA)

sapply(comb_tax, function(x) sum(is.na(x))) -> unassigned_comb_tax
comb_tax_df <- as.data.frame(unassigned_comb_tax)

comb_tax_df$Classifier <- c("RDP","SINTAX","UTAX","COMBINED","RDP","SINTAX","
UTAX","COMBINED", "RDP","SINTAX","UTAX","COMBINED","RDP","SINTAX","UTAX","COM
BINED", "RDP","SINTAX","UTAX","COMBINED","RDP","SINTAX","UTAX","COMBINED", "R
DP","SINTAX","UTAX","COMBINED")

comb_tax_df$Rank <- row.names(comb_tax_df)
comb_tax_df$Assigned <- sqrt((comb_tax_df$unassigned_comb_tax -nrow(comb_tax)
)^2)
comb_tax_df
```

```
comb_tax_df$Classifier <- factor(comb_tax_df$Classifier, levels = c("RDP","UT
AX","SINTAX","COMBINED"))

pdf("outputs/TaxonomicAssignmentComparison_plot.pdf")
ggplot(comb_tax_df, aes(x = Rank, y = Assigned, fill= Classifier)) +
  geom_bar(stat = "identity") +
  scale_x_discrete(limits=comb_tax_df$Rank) +
  theme(axis.text.x = element_text(angle = 90, hjust = 1),
        panel.grid=element_blank(),
        panel.background=element_blank()) +
  ggtitle("Taxonomy Assignments Comparison") +
  labs(x="Taxonomic Ranks", y="Number of classified OTUs") +
  theme(axis.text.x = element_text(vjust=0.5, size=8)) +
  theme(axis.text.y = element_text(hjust=0.5, size=8)) +
  theme(plot.title = element_text(size = 15, face = "bold", hjust = 0.5))
dev.off()
```

Step 4: Troubleshooting.

That's it, you're done!

You will know *constax.sh* is running because it writes each step onto the screen. With the test dataset of 500 OTUs, it will take about 10 minutes to train the classifiers, and generate the taxonomies. If running CONSTAX for multiple datasets, please note, it will write over the contents of *taxonomy_assignments* and *outputs* directories with each run. If users would like to save these files we recommend making a copy of the directory before rerunning CONSTAX.

```
mkdir taxonomy_assignments_test1
cp taxonomy_assignments/* taxonomy_assignments_test1/.

mkdir outputs_1
cp outputs/* outputs1/.
```

Interested in modifying CONSTAX?

Please see descriptions of all custom python scripts implemented in CONSTAX. We have included these descriptions in anticipation that users would like to use CONSTAX with other reference databases. All of the scripts described below can be found within the *scripts* directory.

FormatRefDB.py

The reference formatting step takes the UNITE general release fasta, and changes the information in the header lines, and generates additional files (for the RDP Classifier) so all three classifier programs begin from a reference that contains an identical number of species. The series of scripts *FormatRefDB.py*, *subscript_fasta_addFullLineage.py*, and *subscript_lineage2taxonomyTrain.py* are all called by the *constax.sh* and *config* files.

ConsensusTaxonomy.py

This is the workhorse script. It takes the classifier-specific taxonomy outputs, combines them using the rules described in the Methods, and generates a new consensus taxonomy table. The output is filtered at the cutoff specified in the *config* file. This script also creates a combined taxonomy table which shows each classifier's output side-by-side with the consensus output.

FormatBLAST.py

We have also included a python script that formats BLAST output to match the format of CONSTAX output (when used with Qiime's *parallel_assign_taxonomy.py*). Although we do not recommend use of BLAST for taxonomy assignment of ribosomal sequences. This script is NOT automated through *constax.sh*.
