# Supplementary figures and images for: CONSTAX: a tool for improved taxonomic resolution of environmental fungal ITS sequences
Source: BMC Bioinformatics. 2017 Dec 6;18:538. doi: 10.1186/s12859-017-1952-x (PMC5719527; doi:10.1186/s12859-017-1952-x)

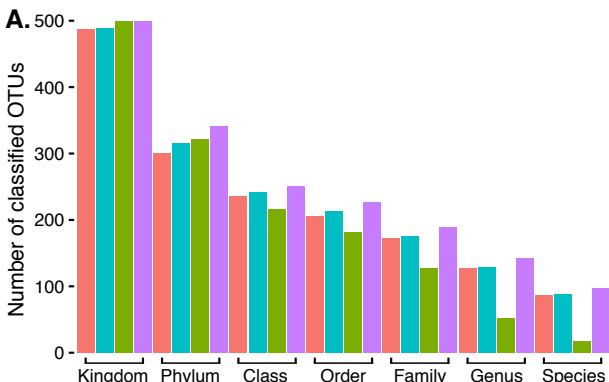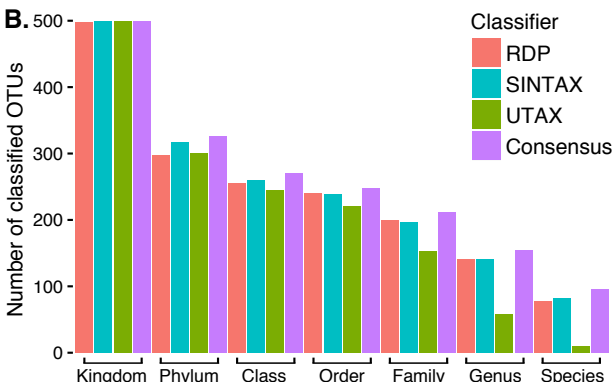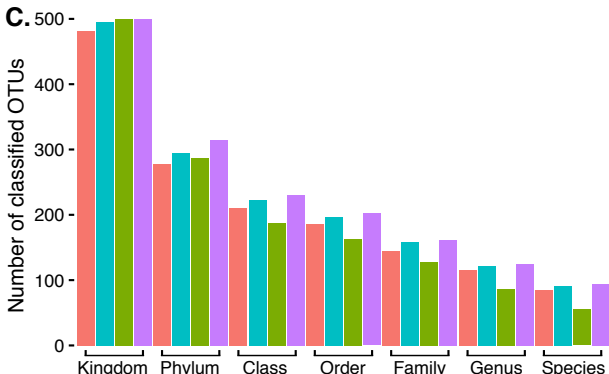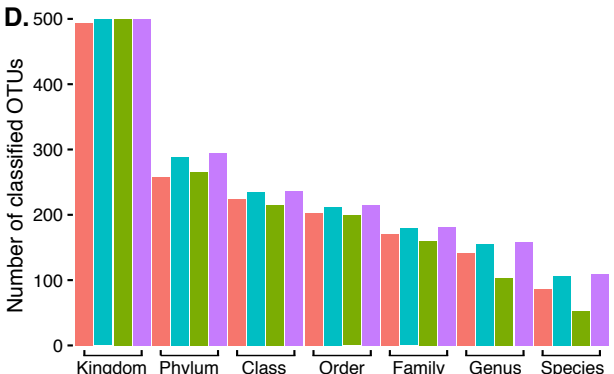

Supplement: Supplementary file 4 — Power of taxonomy classifiers. Distribution of classified and unclassified OTUs for each classifier and across taxonomic level. (A) ITS1-UN and (B) ITS2-UN data analyzed using UNOISE. (C) ITS1-BC and (D) ITS2-BC data analyzed with UPARSE. (PDF 304 kb) [file 12859_2017_1952_MOESM4_ESM.pdf]
